# Cooking as a Health Behavior: Examining the Role of Cooking Classes in a Weight Loss Intervention

**DOI:** 10.3390/nu12123669

**Published:** 2020-11-28

**Authors:** Mattie Alpaugh, Lizzy Pope, Amy Trubek, Joan Skelly, Jean Harvey

**Affiliations:** 1 Department of Nutrition and Food Sciences, University of Vermont, Burlington, VT 05403, USA; efpope@uvm.edu (L.P.); Amy.Trubek@uvm.edu (A.T.); Jean.Harvey@uvm.edu (J.H.); 2 Department of Medical Biostatistics, University of Vermont, Burlington, VT 05403, USA; Joan.Skelly@uvm.edu

**Keywords:** weight management, obesity, diet quality, cooking intervention, food choices

## Abstract

Americans are cooking fewer meals at home and eating more convenience foods prepared elsewhere. Cooking at home is associated with higher quality diets, while a reduction in cooking may be associated with increases in obesity and risk factors for chronic disease. The aims of this study were to examine cooking as an intervention for weight control in overweight and obese adults, and whether such an intervention increases participants’ food agency and diet quality. Overweight and obese adults were randomized into one of two intervention conditions: active or demonstration. Both conditions received the same 24-week behavioral weight loss intervention, and bi-weekly cooking classes. The active condition prepared a weekly meal during a hands-on lesson, while the demonstration condition observed a chef prepare the same meal. The active condition lost significantly more weight at six months compared with the demonstration condition (7.3% vs. 4.5%). Both conditions saw significant improvements in food agency scores and Healthy Eating Index scores, though no significant differences were noted between groups. The addition of active cooking to a weight management intervention may improve weight loss outcomes, though benefits in diet quality and cooking behaviors may also be seen with the addition of a demonstration-only cooking intervention.

## 1. Introduction

Over the last twenty years, Americans’ eating habits have shifted, with fewer meals cooked at home and more meals eaten outside the home from restaurants, convenience stores, fast food locations, and cafeterias [1,2,3]. Because food away from home is typically far less healthy than food eaten at home, it is no surprise that many public health interventions have focused on attempting to make meals away from home healthier [4,5,6,7,8]. However, Americans still report spending more of their disposable income on food eaten at home, and at least 90% report at least sometimes cooking at home [9]. Regardless, it is clear that with innumerable options available to outsource cooking, this once necessary domestic behavior has declined in recent years in step with the rise of convenience food consumption [10]. In fact, the decline in cooking has been cited as being responsible for the increase in the prevalence of obesity and other chronic disease risk factors [11].

Furthermore, evidence suggests that adults who cook dinner frequently at home have diets lower in total energy, fat, and sugar than those who cooked less frequently at home [12]. In addition to research associating cooking at home with better diet quality, there is also evidence that increasing one’s cooking knowledge and/or skill is associated with increased healthy food intake [13,14]. Finally, some research has associated cooking at home with lower Body Mass Index (BMI) [15]. However, the relationship between cooking at home and improved diet quality and weight management is equivocal, with several studies also reporting no relationship between cooking at home and improved health outcomes [16,17,18,19].

Methodological limitations may explain some of these contrary results [20,21]. In many studies, cooking skills or cooking “classes” are rarely presented as a sole component in interventions but are combined with nutrition, exercise, mindfulness, or parenting topics [22]. It is not clear then if cooking has value alone, or just in concert with other treatment components. Current cooking interventions are also quite variable in length, dose, and the level of engagement participants have with hands on cooking; some simply observe skills, while others practice skills and create full meals. Finally, most evaluations of cooking interventions have relied on self-report measures that are rarely validated, thus calling into question the many positive outcomes cited above [21]. 

As stated previously, ready-to-eat, convenience, and restaurant foods are often significantly higher in calories, fat, salt, and sugar than whole, home-prepared foods, yet current state-of-the-art obesity treatment programs do not address cooking or home food preparation skills at all [7]. To our knowledge, there has never been a weight management intervention that includes and isolates a cooking intervention. Yet, adding cooking as a health behavior to a well-proven behavioral obesity treatment may improve weight loss, weight maintenance, and diet quality. 

Therefore, the aims of this study were to determine if the addition of cooking classes to a standard behavioral weight loss program would improve weight loss and diet quality when compared to a weight loss program with an attention only control. A secondary aim was to evaluate changes in food agency and reported cooking frequency between the two conditions. 

## 2. Materials and Methods

### 2.1. Participants

Overweight and obese adults >18 years with a body mass index (kg/m^2^) between 25 and 50 were recruited to participate. Exclusion criteria included a history of major medical or psychiatric conditions; recent changes in medication known to affect weight; current, planned, or recent pregnancy; medical conditions that would exclude exercise; a schedule that would restrict attendance at weekly group meetings; and reported cooking of more than 3 meals at home per week. Recruitment was conducted from October 2018 to March 2019 and was approved by the Committee on Human Research in the Behavioral Sciences at the University of Vermont, ID: 19-0131. The study was registered with ClinicalTrials.gov, registration number: NCT03783962. 

### 2.2. Screening Procedures

Interested individuals applied to participate via a study recruitment website that assessed reported body weight and height information and treatment meeting availability. Informed consent was obtained at the in-person screening and orientation visit. Two trial waves were recruited that each included an active condition and demonstration condition, with 11 to 17 individuals in each group. Enrolled subjects were randomized to active condition groups or demonstration condition groups by a random number randomization scheme. 

### 2.3. Behavioral Weight Control Intervention

All groups received a 24-week manualized comprehensive behavioral weight loss program that met weekly in face to face groups. Participants received identical behavioral lessons and individualized counselor feedback on progress toward meeting exercise and dietary intake goals. The counselor, a Master’s level Registered Dietitian, used a written protocol that outlined standard lessons with counselor guides to ensure the consistency of the intervention. The counselor facilitated the in-person meetings and provided feedback on the weekly self-monitoring journals and homework. 

The weight loss treatment program focused on the modification of eating and exercise habits through the use of behavioral strategies and self-management skills. Participants were prescribed a calorie restricted diet and given a dietary fat goal corresponding to ≤25% of calories from fat. Graded exercise goals were provided which progressed to 200 min/week of moderate to vigorous exercise like walking. Behavioral strategies included self-monitoring, stimulus control, problem solving, goal setting, relapse prevention, and assertiveness training [23,24,25,26]. Homework assignments corresponding to these strategies were provided weekly. Subjects were instructed to record their dietary intake, minutes of physical activity, and weight daily on My Fitness Pal and entries were monitored weekly. All groups met weekly for an hour-long session; therefore, the schedule of contact was the same for both conditions. Meetings were not held on holidays. Therefore, during holiday weeks, participants did not attend in-person meetings, though they were expected to complete daily food logs. Wave 1 of the study included one holiday week and wave 2 included two holiday weeks.

### 2.4. Cooking Intervention

Active Intervention. The active intervention was based on the concept of Food Agency developed by Trubek et al. [27]. Food agency looks at cooking as more than just a manual skill—it takes into account the sensory, sociocultural (e.g., time, money), and physical environments involved in cooking and it incorporates the ability to adapt. Those with high food agency feel empowered in their cooking practice, as they have the planning and preparatory skills, as well as the cooking skills, to be successful [27]. Trubek et al. have developed a pedagogy for cooking classes designed to increase food agency by addressing the cognitive, technical, and mechanical skills necessary to make a meal [20,27]. By using pedagogy designed to increase one’s food agency, the participant is better prepared to overcome potential daily challenges that could prevent them from meeting their cooking, nutrition, and social goals. The pedagogy emphasizes not just skill building like how to read and use a recipe, but adaptability and the development of decision-making and organizational skills that will be helpful no matter the food environment, time, or resource limitations [20]. The concept of food agency views cooking behavior in a more holistic way. It is not enough merely to teach someone the mechanics of cooking. In order for cooking to become an ingrained practice, you must teach someone how to overcome barriers and adapt to the conditions they face in daily life.

The classes are built around helping participants achieve three “understandings”. First, is that sensory analysis done thoughtfully allows for deep engagement in meal preparation and is a means for continued learning and improvement. Second, participants realize that knife skills are essential for transforming raw ingredients into cooked ingredients. Third, the essential function of mise en place (organization of thought, space, tools, ingredients, and process) is important for successfully cooking a meal. Each week, the classes were built around these understandings by focusing on a different skill or part of a meal. Sensory analysis, knife skills, and mise en place were introduced, and then, labs focused on using these skills while learning the basic principles for cooking vegetables, grains, proteins, and a full meal. Classes began with a brief lecture on the day’s topic, followed by a laboratory session. Participants worked in teams of two in the University foods lab to actively practice skills and cook a meal. Meals prepared in class were primarily dinner recipes, with the exception of one breakfast recipe. Recipes were low in calories and fat with a limit of 350 calories per serving and no more than 15 g of fat per serving. Subjects were given recipes and information sheets that covered pantry supplies, grocery lists, knife skills, and cooking equipment. Classes were taught by a chef-educator trained in the pedagogy by Dr. Trubek, and participants had the opportunity to practice sensory analysis and sample the food they prepared at the end of class. Twelve cooking classes were run every other week before the in-person weight loss meetings. 

Demonstrations. The demonstration condition served as an “attention only” control. Previous research suggests that demonstrations of cooking have little to no impact on cooking behavior; therefore, cooking demonstrations were used to “even out” the time and attention devoted to the active cooking participants without introducing bias into the study design [28,29]. Subjects in the demonstration condition also began with a brief lecture on the day’s lesson followed by a cooking demonstration that covered the same topics as the active intervention group. All participants received the same printed information and also had an opportunity to practice sensory analysis and sample the prepared food at the end of class. The demonstrations were led by the same chef as the active intervention group. 

### 2.5. Dependent Measures

All outcomes were assessed at baseline and 6 months, unless stated otherwise. 

Body weight. Weight change from baseline was the primary dependent measure. Weight was taken at baseline, 3 months, and 6 months and was measured in street clothes, without shoes, on a calibrated scale. Height was measured using a wall-mounted stadiometer (Seca Corporation, Hanover, MD, USA). BMI was calculated as weight (kg)/height (m^2^). The percentage of weight loss from baseline was calculated for the 3- and 6-month assessments. Two dichotomous variables were created: weight loss greater than or equal to 5% (<5% vs. ≥5%) and weight loss greater than or equal to 10 percent (<10% vs. ≥10%).

Dietary intake. Dietary intake was measured with the Automated Self-Administered 24-h Dietary Assessment Tool (ASA24). ASA24 is a web-based dietary assessment tool developed by the National Cancer Institute that allows the collection of multiple, automatically coded, self-administered 24-h recalls. ASA24 has been validated and used in many different populations and nutrition studies [30]. Participants were asked to log 3 days of food (2 weekdays and 1 weekend day) at the baseline and 6-month assessment points. 

Healthy Eating Index (HEI). The HEI is a measure of overall diet quality, and can assess compliance with the U.S. Dietary Guidelines, as well as measure changes in dietary patterns. The HEI is updated with each set of Dietary Guidelines, and has been validated for the 2005 and 2010 Dietary Guidelines [31]. The HEI is a valid and reliable tool for assessing dietary quality in a variety of population subgroups and nutrition interventions [31]. The HEI calculates final scores from 24-h recalls like ASA24. 

Cooking and Food Provisioning Action Scale (CAFPAS). The CAFPAS scale, designed to measure food agency, evaluates cooking and food preparation practices or the degree to which individuals can set and achieve cooking and food provisioning goals. The CAFPAS includes 28 items with three subscales: Food Self-Efficacy, Food Attitude, and Structure. The CAFPAS scale has been shown to predict reported meals cooked per week and has adequate internal validity and test–retest reliability [32].

Cooking Perceptions, Attitudes, Confidence, and Behaviors Survey. The Cooking Perceptions, Attitudes, Confidence, and Behaviors Survey is a 53-item survey designed to assess three factors: perceptions of cooking, cooking confidence and attitudes, and cooking behaviors [33]. Questions regarding cooking frequency from the cooking behavior subscale were used to evaluate the number of meals cooked during the previous week. 

Treatment Engagement. Participant attendance at each session was recorded. Completion of self-monitoring journals was assessed weekly. Journals were coded as “complete” if at least 5 of 7 days were recorded, “partial” if 1 to 4 days were entered, and “none” if no days were completed. The counselor also recorded if weekly calorie and exercise goals were met.

Cooking class evaluation. The cooking class evaluation asked participants to assess their impressions of the cooking classes, chef-educator, recipes, and perceived changes to their cooking and health behaviors as a result of participating in the classes. Twenty-three statements were included in the survey, and each category of questions contained between 3 and 7 statements. Participants selected their level of agreement with statements on a seven-point Likert scale, where 7 indicated strongly agree, 1 indicated strongly disagree, and 4 indicated neutral. Evaluations were completed online following the final cooking class meeting.

### 2.6. Statistical Analysis

All analyses were performed using SAS Version 9 statistical software (SAS Institute, Cary, NC, USA) unless noted otherwise. Demographic characteristics were compared between treatment conditions using *t*-tests for continuous measures and chi-square tests for categorical variables. Fisher’s exact test was used to compare treatment groups on percentage of participants reporting weight at all three assessments.

Repeated measures analysis (SAS, PROC MIXED) was used to assess differences between groups in mean weight across assessments. Linear contrasts were constructed to compare the treatment groups on the magnitude of weight change from baseline to 3 and 6 months. Group differences in the percentage of weight loss from baseline at 3 and 6 months were assessed using a repeated measures analysis. Repeated measures for categorical data based on generalized estimating equations (GEE) utilizing a logistic link function (SAS PROC GENMOD) were used to compare treatment groups on weight loss ≥5% across assessments. No women achieved ≥10% weight loss by 3 months, so a chi-square test was used to compare treatment groups at 6 months.

The percentage of classes attended and journaling compliance were compared between treatment groups using Wilcoxon Rank Sum tests. Repeated measures analysis was used to assess group differences over time in the Healthy Eating Index scores, Cooking and Food Provisioning Action scales, and the average number of meals cooked at home. Means were calculated for scale-aligned class evaluation data and were compared between treatment conditions using two sample *t*-tests with IBM SPSS statistics for Windows, Version 26.0 (IBM Corp, Armonk, NY, USA).

## 3. Results

### 3.1. Participants

**Figure 1:**
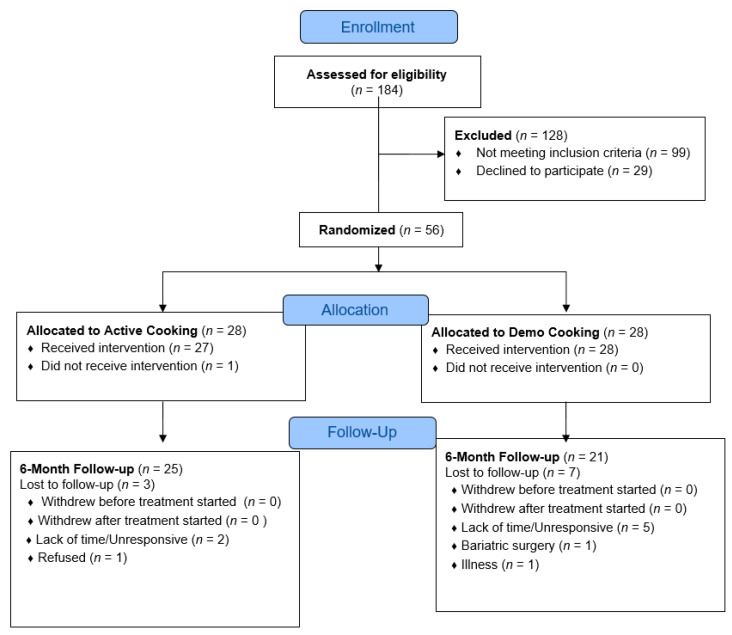
Consort flow diagram.

A total of 56 participants were randomized into one of two conditions: active cooking or demonstration. (Figure 1). Participants were predominantly non-Hispanic white, female (89%), and well-educated, with 73% reporting a college or graduate degree. There were no significant baseline differences between conditions with respect to sociodemographic characteristics (Table 1). At baseline, participants in the active condition had lower body weight and BMI on average compared with participants in the demonstration condition, though these differences were not significant. Participants were randomized in two waves. 

Follow-up weight and BMI data were provided by 94.6% of randomized participants at 3 months, and 89% at 6 months. Weight data reporting trended higher for participants in the active condition at 3 and 6 month follow-ups (96% at both time points) when compared with the demonstration group at 3 and 6 months (93% and 82%, respectively), though these differences were not statistically significant.

### 3.2. Weight Change

There was no significant difference in weight loss between conditions at 3 months (Table 2). However, participants in the active condition lost significantly more weight at 6 months than those in the demonstration condition (7.34 ± 0.63 vs. 4.49 ± 0.67 kg, *p* < 0.03). The conditions also differed significantly in the mean percentage of initial body weight lost at 6 months, with the active condition losing a greater percentage of their body weight compared with the demonstration condition (*p* = 0.001). A greater portion of active condition participants (67%) experienced clinically significant weight loss (≥5% weight loss) at six months compared with 43% of the demonstration condition, though this difference was not statistically significant (*p* = 0.10). 

### 3.3. Treatment Engagement 

Treatment engagement, as measured by class attendance and journal submissions, trended higher in the active condition group compared with the demonstration condition group. Compliance with class attendance was expressed as a percentage of classes out of a possible 24 that each participant attended. Likewise, journaling compliance was expressed as a percentage of weeks participants submitted complete or partial food journals out of a possible 25 weeks for those in wave 1 or 26 weeks for those in wave 2. During the 6-month study, the median percentage of scheduled classes attended by participants in the active group was 83% (Interquartile range (IQR): 77–87), while the demonstration group attended 69% (IQR: 29–83) of scheduled classes (*p* = 0.009). Recorded weekly journal compliance trended higher in the active group (96%, IQR = 72–100) when compared with the demonstration group (88%, IQR = 35–98), though this difference was not statistically significant (*p* = 0.12).

### 3.4. Diet Quality

Healthy Eating Index (HEI) scores were significantly higher for both groups at 6 months when compared with baseline measures (Table 3). There was no significant difference between groups at baseline or 6 months.

### 3.5. Food Agency

Participants in both groups had a significant increase in total CAFPAS scores and two of the three food agency subscales (self-efficacy and attitude) from baseline to 6 months (Table 4). The active group scores trended higher for each subscale and total score at baseline, though these differences were not significantly different from the demonstration group.

### 3.6. Cooking Behavior

Participants in the demonstration condition reported cooking significantly more meals (breakfast, lunch, and dinner) at home each week at 6 months compared with baseline (Table 4). In comparison, participants in the active condition reported cooking significantly more lunches at 6 months compared with baseline, while the group change in number of dinners cooked was nearly significant (*p* = 0.05) and the change in breakfasts cooked was not significant. Only change in the number of lunches cooked was significantly different between groups. 

### 3.7. Class Evaluations

On average, participants in both groups agreed with statements that reflected positive impressions of the cooking intervention (Table 5). When compared with the active group, the demonstration group was significantly more likely to strongly agree with positive statements overall (6.42 vs. 6.64, *p* = 0.03).

## 4. Discussion

To our knowledge, this is the first study to include an active cooking intervention with a weight management intervention. The novel addition of active cooking to a weight management intervention significantly increased weight loss over six months compared with the addition of a demonstration-only cooking intervention. Mean body weight loss in the active condition was 3.5 percentage points greater than mean body weight loss for the demonstration-only condition. Furthermore, weight loss observed in the active cooking condition met or exceeded six-month averages in comparable weight loss interventions that did not include a cooking component [34,35,36]. When compared with similar weight management studies in the literature, adding demonstration cooking classes to a weight-loss intervention did not improve weight loss. In fact, mean six-month weight loss in the demonstration condition was lower than what has been observed in similar studies [34,35,36]. The data suggest that the addition of an independent active cooking component to a weight-loss intervention significantly improved weight loss outcomes, while a demonstration-only cooking intervention did not.

In addition to losing more weight, participants in the active condition also had significantly higher class attendance for both the cooking class and weight loss class compared with participants in the demonstration condition. The correlation between greater weight loss and higher class attendance is consistent with research by Burke et al., which found that greater compliance in terms of class attendance tends to predict greater weight losses [37]. Additionally, self-monitoring using food journals, also correlated with greater weight loss, trended higher in the active cooking condition, though was not significantly greater when compared with the demonstration condition [38,39]. Perhaps a larger sample size would enable us to detect a significant difference between conditions.

Surprisingly, there was no difference in diet quality, as measured by HEI scores, between the two conditions, despite the active condition experiencing greater weight loss. Though both groups saw significant improvements in their HEI scores at 6 months, this does not help to explain why the active condition experienced significantly more weight loss when compared with the demonstration group. A possible limitation of using HEI scores to explain weight loss or differences in BMI is that HEI scores do not account for the appropriateness of energy intake [40]. Therefore, it is unclear if the meals reported by participants in either group met or exceeded their calorie goals. 

Despite a significant difference in weight loss, there was no significant difference in food agency measures between groups. However, both groups demonstrated a significant increase in CAFPAS scores from baseline to 6 months, suggesting that food agency is enhanced with the addition of a cooking intervention, whether active or demonstration. It was expected that taking part in active cooking classes would result in higher food agency compared with demonstration-only cooking classes. 

Mean baseline CAFPAS scores of 11.25 ± 0.37 and 10.41 ± 0.37 for the active and demonstration conditions, respectively, were well below the average score of 15.4 reported in previous research for a group demographically similar in race, education, and age [32]. Though scores increased significantly for both groups after 6 months of active or demonstration interventions (13.3 ± 0.40 and 13.4 ± 0.41, respectively), they remained below the sample average found by Lahne et al. [32]. The low baseline CAFPAS scores may have provided enough room for improvement such that participants were able to see positive effects even when provided only demonstration classes. 

Increased CAFPAS scores paralleled an increased number of meals cooked at home for both conditions. In fact, both conditions’ weekly cooking frequency totals increased more than would be expected based on changes in CAFPAS scores from baseline to 6 months. Previous research found an increase of 1 point in total CAFPAS score predicted an increase of 0.96 meals cooked at home per week, while this study found an increase of 1 point in total CAFPAS score predicted a 1.1 and 1.57 meal increase for the active and demonstration conditions, respectively [32]. This finding may be a result of comparing a change in CAFPAS scores over time for the same individual, rather than single time point scores compared between individuals, as was the case in Lahne et al. [32]. We hypothesized that the active cooking group would have greater cooking frequency at home, thus greater weight loss. Surprisingly, however, it was the demonstration group whose cooking frequency increased by more than twofold over the active cooking group, despite weight losses not being as robust. 

It is important to highlight that demonstration classes based on the food agency pedagogy appear to confer benefits in terms of diet quality and cooking behaviors, even in the absence of weight loss. Additionally, the data suggest that participants in the demonstration group found the cooking classes as or more enjoyable and valuable than participants in the active group. These may be useful findings for health education initiatives that must consider cost and space limitations in program planning. Demonstration-only cooking classes require less resources by way of food purchasing, setup and cleanup support, and necessary space and equipment, all of which may be hurdles to implementing cooking education programs. Though class structure differed by hands-on practice versus observation, it is important to note that all participants had the opportunity to participate in a sensory analysis and eat the prepared meal. Therefore, sensory analysis may be a critical component of cooking interventions aimed at improving food agency and cooking behaviors. 

This study has several strengths, including a randomized design that included an evidence-based behavioral weight loss program common to both conditions [35,36,41]. A registered dietitian with several years of experience in weight management led the behavioral weight management classes for all groups; likewise, a chef-educator trained in the food agency pedagogy taught all cooking classes for both conditions. Differences between cooking conditions were further controlled by implementing uniform class duration, setting, recipes, and lessons, leaving the only distinction between conditions to be delivery type (active or demonstration). Additional strengths include objective measures of weight being collected and reliable tools being used to gather diet quality and food agency scores. 

The small sample size of the study may have limited power in determining statistical significance for some tests. Study sample size was determined by the capacity of an eight-station kitchen lab, class times convenient for participants, and times that the lab space was available. Increasing the sample size for a similar study would require a larger kitchen space or an extended timeframe, both of which would significantly increase the cost burden. 

Additionally, the data represent a 6-month study window and do not offer insight into sustained changes over time. The generalizability of the study to the broader overweight and obese population may be limited by certain demographic factors of the study population, including a low level of racial diversity, high education level, and few male participants.

## 5. Conclusions

The findings of this study indicate that the addition of an active cooking intervention to a behavioral weight loss program may be an effective way to increase weight loss outcomes. Though there did not appear to be any weight loss benefits to including a demonstration-only cooking intervention, participants in the demonstration condition saw significant improvements in food agency, diet quality, and cooking frequency at six months. 

## Figures and Tables

**Table 1:** Baseline characteristics of participants.

	Active	Demonstration	*p*-Value
	*n* = 28	*n* = 28	
Age, years (mean ± SD)	55 ± 11	50 ± 11	0.12
Female (*n*; %)	25 (89%)	25 (89%)	1.0
Non-Hispanic White (*n*; %)	28 (100%)	24 (86%)	0.11
Education (*n*; %)			
HS grad/GED/Some college	8 (28%)	7 (25%)	0.35
College degree	10 (36%)	15 (54%)	
Grad/Prof Ed.	10 (36%)	6 (21%)	
Work for pay full-time (*n*; %)	22 (78%)	21 (75%)	0.75
Married (*n*; %)	16 (57%)	17 (61%)	0.79
Weight, pounds (mean ± SD)	204.2 ± 35.9	216.5 ± 45.1	0.26
BMI at baseline (kg/m^2^) (mean ± SD)	33.9 ± 5.8	35.5 ± 5.7	0.31

**Table 2:** Mean weight loss from baseline to three months and six months.

	3 Months from Baseline			6 Months from Baseline		
	Active *n* = 27	Demonstration *n* = 26	*p*-Value	Active *n* = 27	Demonstration *n* = 23	*p*-Value
Weight loss (kg) (LSMean ± SE)	3.27 ± 0.63	2.83 ± 0.64	0.63	7.34 ± 0.63	4.49 ± 0.67	0.003
Percent Weight loss (LSMean ± SE)	3.65 ± 0.72	2.83 ± 0.73	0.42	8.30 ± 0.72	4.79 ± 0.76	0.001
Percent losing ≥ 5%	25.93	19.23	0.56	66.67	43.48	0.10
Percent losing ≥ 10%	0%	0%		29.63	17.39	0.32

**Table 3:** Mean healthy eating index scores from baseline to six months.

	Baseline	6 Months	Change from Baseline to 6 Months	Time *p*-Value	Group × Time *p*-Value
Active (LS Mean ± SE)	*n* = 28	*n* = 23			0.97
	54.93 ± 2.19	61.01 ± 2.17	6.08 ± 2.62	0.02	
Demonstration (LS Mean ± SE)	*n* = 28	*n* = 18			
	52.05 ± 2.19	58.28 ± 2.44	6.23 ± 2.84	0.03	

**Table 4:** Mean food agency scores (CAFPAS) and mean meals cooked at home per week from baseline to six months.

	Active Group			Demonstration Group			Group × Time *p*-Value
	Baseline *n* = 28	6 Months *n* = 23	Time *p*-Value	Baseline *n* = 28	6 Months *n* = 19	Time *p*-Value	
CAFPAS Scores							
Self-efficacy	3.59 ± 0.15	4.65 ± 0.17	<0.001	3.35 ± 0.15	4.70 ± 0.18	<0.001	0.25
Attitude	4.45 ± 0.17	5.31 ± 0.18	<0.001	4.32 ± 0.17	5.37 ± 0.19	<0.001	0.40
Structure	3.20 ± 0.19	3.37 ± 0.20	0.40	2.74 ± 0.19	3.32 ± 0.21	0.01	0.18
Total	11.25 ± 0.37	13.34 ± 0.40	<0.001	10.41 ± 0.37	13.40 ± 0.41	<0.001	0.08
Average meals cooked at home in the past week							
Breakfast	2.46 ± 0.37	2.82 ± 0.41	0.41	1.54 ± 0.37	2.59 ± 0.43	0.02	0.26
Lunch	1.07 ± 0.34	2.17 ± 0.38	0.02	1.07 ± 0.34	3.58 ± 0.40	<0.001	0.03
Dinner	2.57 ± 0.33	3.37 ± 0.36	0.052	3.25 ± 0.33	4.38 ± 0.39	0.011	0.58

Note: Tables values represent Least Square Means ± SE.

**Table 5:** Cooking class evaluation statements and group Likert scale means.

	Active Group	Demonstration Group	*p* Value
The cooking classes…	(*n* = 23)	(*n* = 20)	
were enjoyable to attend.	6.83	7.00	0.04
were too short to cover the material and prepare a meal. ^+^	6.00	6.11	0.71
introduced me to foods I had not cooked before.	6.65	6.80	0.56
introduced me to cooking techniques I had not used before.	6.43	6.80	0.17
helped me improve my cooking skills.	6.52	6.70	0.45
will NOT affect how I cook at home. ^+^	6.35	6.50	0.48
will support me in achieving healthy eating goals.	6.43	6.40	0.90
The chef instructor was…	(*n* = 23)	(*n* = 20)	
knowledgeable about the material and able to answer questions.	7.00	6.95	0.33
enthusiastic and made the lessons fun.	7.00	6.95	0.33
clear and easy to understand.	7.00	6.95	0.33
The recipes used in class…	(*n* = 23)	(*n* = 20)	
were enjoyable to make and eat.	6.39	6.80	0.06
were useful for me to take home and use again.	5.96	6.65	0.03
helped me learn skills that I can use when preparing other dishes.	6.65	6.70	0.79
Having taken the cooking classes I am…	(*n* = 23)	(*n* = 20)	
more likely to cook using varied or different ingredients than before the class.	6.48	6.65	0.40
more likely to repeat the recipes I learned in class.	6.48	6.65	0.40
NO more likely to try new or unfamiliar recipes. ^+^	5.82	6.63	0.05
more likely to pay more attention to the food I eat	5.87	6.35	0.12
NO more likely to use spices, herbs, and other condiments to bring more flavor to my meals. ^+^	6.22	5.85	0.45
more likely to cook meals that I consider healthy	6.22	6.55	0.12
The cooking lessons…	(*n* = 23)	(*n* = 19)	
helped me understand new cooking techniques and how to handle ingredients.	6.57	6.74	0.33
covered appropriate introductory cooking skills.	6.65	6.79	0.34
were hard to follow and keep up with. ^+^	6.70	6.63	0.72
helped me succeed in losing weight	5.43	5.37	0.89
Total Group Means	6.42	6.64	0.03

^+^ Likert scale was reverse coded to calculate means.
